# Lack of informed consent for surgical procedures by elderly patients with inability to consent: a retrospective chart review from an academic medical center in Norway

**DOI:** 10.1186/s13037-019-0205-5

**Published:** 2019-06-22

**Authors:** Jorgen Dahlberg, Vegard Dahl, Reidun Forde, Reidar Pedersen

**Affiliations:** 1 Division of Surgery/Centre for Medical Ethics, University of Oslo, Faculty of Medicine, Akershus universitetssykehus, 1478 Lørenskog, Norway; 2 Division of Surgery, University of Oslo, Faculty of Medicine, Akershus universitetssykehus, 1478 Lørenskog, Norway; 3 Centre for Medical Ethics, University of Oslo, Faculty of Medicine, Kirkeveien 166, Fredrik Holsts hus, 0450 Oslo, Norway

**Keywords:** Informed consent, Competent, Competence, Autonomy, Coercion, Patient rights, Legal requirements

## Abstract

**Background:**

Respect for patient autonomy and the requirement of informed consent is an essential basic patient right. It is constituted through international conventions and implemented in health law in Norway and most other countries. Healthcare without informed consent is only allowed under specific exceptions, which requires a record in the patient charts. In this study, we investigated how surgeons recorded decisions in situations where the elderly patient’s ability to provide a valid informed consent was questionable or clearly missing.

**Method:**

We investigated all medical records of patients admitted to surgical departments in a Norwegian large academic emergency hospital over a period of 38 days (approximately 5000 patients). We selected records of patients above the age of 70 (570 patients) and searched through these 570 medical records for any noted clear indications of inability to consent such as “do not understand”, “confused” etc. (102 patients). We read through all the medical records on these 102 patients noting any recordings on lack of informed consent, any recordings on reasoning and process hereto. We also took note whether there were clear indications on the use of coercion.

**Results:**

None of the 102 included patients´ charts contained legally valid recorded assessments (for example related to the patients´ competence to consent) when patients without the ability to consent were admitted and provided healthcare.

Some charts contained records that the patient resisted treatment, thus indicating treatment with coercion. In these situations, we did not find any documentation related to legal requirements that regulate the use of coercion.

**Discussion and conclusion:**

We found a substantial lack of compliance with the legal requirements that apply when obtaining valid informed consent. There are many possible reasons for this: Lack of knowledge of the legal requirements, disagreement about the rules, or that it is simply not possible to comply with the extensive formal and material legal requirements in clinical practice. The results do not point out whether the appropriate measures are amending the law, educating and requiring more compliance from surgeons, or both.

## Background

Historically healthcare has, to a large extent, been based in a paternalistic approach where healthcare personnel assesses what is in the patients´ best interest. The focus on respecting the patients´ autonomy has increased significantly during the last decades [1]. Healthcare legislation is now to a much larger degree based on providing healthcare adapted to the individual patient’s preferences [2, 3]. This is operationalized through the informed consent, nationally in the Norwegian Patients´ Rights Act Article 4–1 from 1999 [4], in international conventions including the Convention on Human Rights and Biomedicine from 1997, Article 5 [5], and through a significant amount of national and international guidelines and directives Table 1.

**Table 1:** Informed consent

The Norwegian Patients´ Rights Act Article 4–1 Informed consent	The Oviedo Convention Article 5 – General rule
Health care may only be provided with the patient’s consent, unless legal authority exists or there are other valid legal grounds for providing health care without consent. In order for the consent to be valid, the patient must have received the necessary information concerning his health condition and the content of the health care.	An intervention in the health field may only be carried out after the person concerned has given free and informed consent to it. This person shall beforehand be given appropriate information as to the purpose and nature of the intervention as well as on its consequences and risks. The person concerned may freely withdraw consent at any time.

Valid consent requires voluntary assent, information, and that the patient is competent to consent. As a general rule healthcare personnel have to establish that the patient has competence with regards to personal-, material- and procedural requirements [6], herein provide adequate information, ask about the patient’s preferences, assess the patients’ competence, and avoid coercion [3].

Exception from this rule must be stated clearly. In surgery, the most common exceptions from the requirement of consent to healthcare are either (i) that the patient lacks the competence to provide a valid consent [7, 8] or (ii) that the vital importance of the situation requires immediate action [9, 10] Tables 2 and 3.

**Table 2:** Exemption to consent upon lacking competence to consent

The Norwegian Patients´ Rights Act Article 4–3 (2) Competence of consent	The Oviedo Convention Article 6 – Protection of persons not able to consent
Competence to give consent may cease to apply wholly or partly if the patient, on account of a physical or mental disorder, senile dementia or mental retardation, is clearly incapable of understanding what the consent entails.	Subject to Articles 17 and 20 below, an intervention may only be carried out on a person who does not have the capacity to consent, for his or her direct benefit. … Where, according to law, an adult does not have the capacity to consent to an intervention because of a mental disability, a disease or for similar reasons, the intervention may only be carried out with the authorisation of his or her representative or an authority or a person or body provided for by law.

**Table 3:** Exemption to consent in emergencies

The Norwegian Health Personell Act Article 7 – Emergency health care	The Oviedo Convention Article 8 – Emergency situation
Health personnel shall immediately provide the health care they are capable of when it must be assumed that the health care is of vital importance. Pursuant to the limitations laid down by the Patients’ Rights Act section 4–9, necessary health care shall be given, even if the patient is incapable of granting his consent thereto, and even if the patients objects to the treatment	When because of an emergency situation the appropriate consent cannot be obtained, any medically necessary intervention may be carried out immediately for the benefit of the health of the individual concerned.

In Norway, like most other countries, the provision of a valid consent or lack of such should be recorded in the patient chart according to the law [11]. When lacking valid consent, any given healthcare requires specific reasons, which also are to be recorded.

We have little knowledge about to what extent ordinary clinical practice complies with these principles and laws. Based on previous research and our own clinical experience, we had reason to suspect that there might be a lack of compliance with these legal requirements in surgery specifically [12–17]. We wanted to investigate whether surgeons assess the patients’ capacity to provide an informed consent where the patient’s ability to provide a valid consent was questionable or clearly missing, and furthermore whether surgeons generally comply with the other legal requirements related to providing healthcare to patients who are not able to provide valid consent. Our main hypothesis was that the clinical practice, to a large extent, does not comply with the legal requirements. A prior PubMed search did not identify any previous studies of surgeons’ compliance with the requirements of assessing competence to consent in our region (Scandinavia).

## Methods

**Fig. 1:**
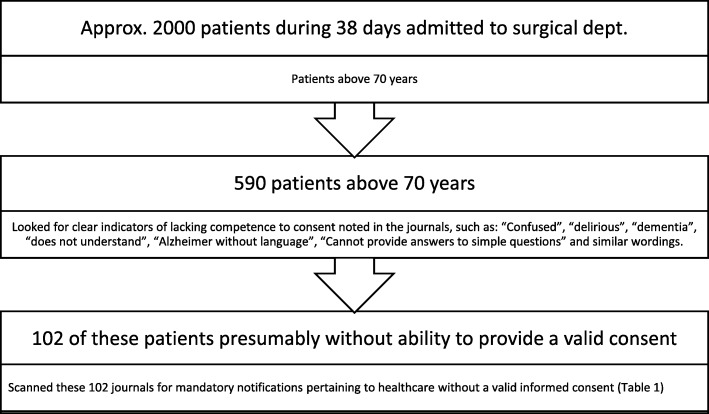
Screened charts with indicators and included patients with the presumed inability to consent

We conducted the study at a large emergency hospital in Norway. Since healthcare without valid consent requires specific documentation in the patient records, we decided to investigate the medical records of all patients admitted to surgical care with a high probability of lacking the competence to provide informed consent. We wanted to include at least 100 patient charts (Fig. 1), which resulted in an inclusion period of 38 days (from September 1st to October 8th, 2013).

We read all the charts of patients above the age of 70 years admitted to the department of surgery which included the units of gastric surgery, breast−/endocrinology surgery, thoracic- and vascular surgery, urology unit, gynecology unit, orthopedic unit and the intensive care unit. The age limit was selected to include patients with a higher likelihood of reduced competence to consent [12, 18]. We screened these charts for noted indicators that the patients lacked the competence to consent during admission and treatment. The condition for competence is generally associated with the requirement that the patient “understands” [7, 13]. We searched specifically for a wording confirming a lack of understanding or competence. We also searched for clinical conditions associated with lacking competence, such as dementia, impaired cognitive function, confusion, or disorientation. We included 102 patient charts Table 2.

We screened these charts reading through all medical records from pre-hospital reference until either the surgical procedure/equivalent treatment was started, or if no such treatment was instigated, to discharge. We noted whether the absence of informed consent was recorded in the charts. We also noted if the reasoning and process hereto were recorded as required according to law.

We specifically noted whether the legal requirements for providing healthcare to a patient lacking the competence to consent were met and recorded by assessing the inclusion of relevant (legal) requirements in each patient chart (Table 4).

**Table 4:** Mandatory notifications about healthcare decisions in lack of competence and/or without a valid informed consent

	Legally required notifications in the medical records (Patients’ Rights Act. art. 4–3, 4–6, chapter 4A, and Health Personnel Act article 7)	NUMBER OF CHARTS (and a short description of relevant notifications found)
1.	Reference to that the decisional competence had been assessed?	NONE
2.	Reference to the lack of ability to provide consent, such as “lack of competence”, “does not understand what the consent entails” or similar wording?	ONE CHART stated “lack competence” but no further notifications or information hereto was included
3.	Noted that the conclusion on lack of decisional competence had been presented to the patient?	NONE
4.	Noted that the conclusion on lack of decisional competence had been presented to the relatives or such other person representing the patient?	NONE
5.	Documentation of information on patient’s preferences with regards to treatment obtained from relatives or another person representing the patient?	NONE
6.	Noted information that the physicians admitting, treating and/or the surgeon operating the patient had conferred with a colleague or other competent health care professionals on the assessment of decisional competence and the reasons for the decision that was made?	NONE
7.	Healthcare provided due to a vital emergency (The Norwegian Health Personnel Act art. 7).	One chart stated “vital indication”
8.	Healthcare provided with the use of coercion (decision provided according to the Patients´ Rights Act chapter 4A, see Table 5)	NONE

Since healthcare in Norway may be provided under certain conditions without consent if the healthcare is of vital importance, we also noted whether there were records that the need for treatment qualified as vital importance Table 3.

Furthermore, we also took note whether there were clear indications on the use of coercion, e.g., documentation of patient resistance. In cases of coercion, we looked for references to the legal requirements for the use of coercion. We specifically searched for whether a formal decision on coercion had been made or whether the use of coercion was justified through the emergency healthcare article. The notifications hereunder were included where it appeared in the charts that the patient showed resistance.

The first author collected the data, where after the last author conducted an internal audit of a blinded and random screening of 10% of the included charts. No substantial deviations were identified in the audit.

## Results

During the inclusion period of 38 days, about 2000 patients were admitted to the surgical departments (see Fig. 1). Five hundred eighty-nine patients were above the age of 70 years, and 102 patients fulfilled the inclusion criteria.

The 102 patients consisted of 58 women and 44 men with a median age of 84 years. Fifty-eight patients were admitted to the orthopedic unit, and 44 were admitted to other surgical units (2 of these patients were admitted to the ICU). Ninety-seven admissions were direct referrals (not previously planned referrals or open returns), and the remaining 5 were previously planned operations or open admissions/returns.

The vast majority of the orthopedic patients were traumatic injuries, such as hip fractures, while the other surgical patients were admitted for various reasons where abdominal-, cardiovascular- and urology-surgery were the most common types of operations.

None of the 102 included charts contained the legally required documentation to provide healthcare in the absence of valid consent from the patient (Table 4).

Of the 102 admissions, 53 contained an operation chart, indicating that 53 out of 102 ended up in surgery. Even though all the included patients most likely were not competent to consent, we found that 13 of the specific operation charts stated that the specific surgical procedure was done with the patient’s informed consent.

The remaining 49 patients without a specified operation chart were, in most cases, treated with other means such as medication or minor surgical interventions (such as catheters inserted not requiring an operation room scheduled). The required documentation lacked for these as well throughout the treatment.

However, one chart did state that the patient lacked competence (without any further records related to this).

One chart contained records stating that healthcare had been required “without information” due to an emergency (“on vital indication”). There was, in other words, only one chart that specifically noted that the reason to treat without consent was a vitally important situation possibly exempting the requirements to assess the competence and provide valid consent. There was no further documentation around the lack of (competence to) consent or reasoning for providing healthcare without such consent in this chart. This case was about a multi-morbid patient without the ability to speak or eat without aid referred to operation for a hip fracture. It is reasonable to assume that this chart also lacked all of the added relevant records.

We found 31 charts in the screened records that reported resistance from the patient, thus indicating that coercion may have been used. Among these, several charts contained clear records on the use of actual coercive measures, such as having inserted intravenous lines on “uncooperative” patients after the patient having physically removed such intravenous lines, use of sedation to get the patient to comply, and physical restrictive measures such as guard rails and hiding medication in food (Table 5).

**Table 5:** Indicating coercion

Among the 31 charts documenting resistance from the patient:	
- 7 noted that the patient removed or tried to remove i.v. lines.	
- 5 specifically noted that sedation was used to circumvent resistance.	
- 3 specifically noted that bed guard rails were used as a restrictive measure.	
- 1 noted that medicine had been concealed in food to get the patient to take medication the patient refused to take.	

## Discussions

We conducted this study in part because we wanted to investigate how successful it is to use legislation to secure patient rights and ethical standards. The results indicate that there are limitations to the effect of such regulations alone. Our findings suggest a clear gap between the legal procedures required and the clinical practice in surgery when a valid consent cannot be obtained.

Furthermore, the regulations on the ethically challenging decisions to use coercion seem to be widely neglected. The use of coercion is subject to extensive regulation both in material conditions and formal requirements specifically because the legislator wishes to emphasize the importance of proper conduct and due processes in these situations. The results raise the question of the effect of using legislation to strengthen patient autonomy.

The findings may partly be explained by a lack of knowledge of the legal requirements and how to apply these in actual clinical practice. It may also be explained by a lack of motivation to conform to the formal procedures (meaning that healthcare personnel do know what a valid consent is and how to go about when it is missing but lack the will to comply with the formal requirements). Finally, there may be external barriers, such as time, or lack of training on how to assess competence to consent. This could also be the case if the findings are merely a result of lacking recordings due to unavailable resources (such as no time or no personnel to handle transcripts). As some charts contain clear indications that valid consent cannot be obtained, but still state that informed consent has been provided, some of these cases are clearly a problem of lacking the knowledge and not only a problem of getting the formalities in order.

The requirements to the recordings will (as stated above) to some extent depend on the emergence of the situation. Even though only one chart contained a reference to that the decision was made in an emergency “on vital indication”, there is a high probability that some of the other cases also pertain to vitally important healthcare exempting the more extensive obligation to assess and provide a valid a consent.

The law does not provide much more clarification on the differentiation between vital important healthcare and other healthcare situations. A certain degree of professional discretion is therefore required in order to assess if the situation is so vital that it exempts the requirements of consent. Even though vital important emergencies allow an exception of assessing autonomy, there are still obligations on how to record these decisions and medical records on such indications of vitality and emergency lack in our study.

It should be noted that a total of 58 patients were orthopedic admissions (and none of them appeared to represent cases with an imminent risk of dying) and 5 admissions were planned/open (presumably not operations due to vital emergencies). The majority of the included charts are presumably not cases of vital emergency, and none of them stated that consent or an assessment of competency could not be obtained due to the emergence of the situation. Therefore, these charts should have contained documentation of the assessment of competence and how the decision was made.

Our primary objective was to study the formal process of evaluating the validity of consent and the process of reaching a decision to provide healthcare where the competence to consent was questionable or clearly lacking. Even though the use of coercion was not a part of our primary objective, the existence of coercion was documented several times. This is especially problematic considering that coercion without clear justification and due process represents a significant infringement in patients’ rights. Our findings clearly indicate that the formal requirement for the use of coercion was largely neglected, and there was little documentation to support the justification of such use.

There is no indication in our data to support that more extensive regulation alone will secure a better practice. It may, on the contrary, be a reason to look at whether not simplifying the rules may serve to improve compliance to the legal requirements and the intentions hereto. Other measures should probably also be considered either in addition to or instead of regulations, such as added education/training, guidelines/procedures, ethics support, etc.

There is no doubt that the results include some cases where there is a lack of compliance with the legal requirements. Regardless of the reasons hereto any breach of legal requirements may result in sanctions from the health authorities, which potentially represents significant challenges to the existing practice that we disclosed.

### Study limitations

This study was conducted on charts, and one should be careful with concluding that this represents the actual clinical practice. However, one of our objectives was also to evaluate compliance with formal requirements and procedures. This also refers to the obligation of providing a record in this regard.

The study has been conducted at one hospital only, and only on geriatric surgical patients. One should be cautious with generalizing the results to other hospitals, departments, medical fields, patient groups, or countries. However, when we have presented these results in Norway, health care professionals have told us that these findings are not unique to this hospital or type of service. Also national numbers on formal decisions on coercion in non-psychiatric hospital wards – with very few formal decisions reported - indicate that the results are not unique to our context [19].

## Conclusion

We found a substantial lack of compliance with the legal requirements that applied when the surgeons provided healthcare to patients that were not able to provide valid informed consent. Our study revealed a fundamental lack in the documentation on elderly patients with reduced competence to consent and the decision-making process in these cases. Our data also point out that there may be significant shortcomings in the decision-making processes related to the use of coercion as well. More generally, the results imply that there may be extensive infringements of patient rights within hospital healthcare.

The results do not point out whether the appropriate measures are amending the law, educating and requiring more compliance from surgeons, or both.

There is a clear need for more research and education in this area, and to further investigate the generalizability of our findings and where the problems reside.

## Data Availability

Data for this study is stored in a secure platform that ensures all transmissions are fully encrypted, end-to-end. The unidentified datasets used for analysis for the current study are available from the corresponding author on reasonable request.

## Abbreviations

I.V.lines: Intra Venous lines
ICU: Intensive Care Unit
